# Case Report: SAPHO syndrome mimicking osseous relapse after haploidentical allogeneic hematopoietic stem cell transplantation for acute myeloid leukemia

**DOI:** 10.3389/fmed.2026.1936597

**Published:** 2026-09-28

**Authors:** Wenhui Zhang, Xiaolin Ma, Xianglan Liu, Chunting Zhao, Hairong Fei, Xue Shi, Wei Wang, Xuewei Li, Xiaodan Liu

**Affiliations:** 1 Department of Hematology, The Affiliated Hospital of Qingdao University, Qingdao, Shandong, China; 2 Department of Bone Marrow Transplantation, The Affiliated Hospital of Qingdao University, Qingdao, Shandong, China; 3 Department of Pathology, The Affiliated Hospital of Qingdao University, Qingdao, Shandong, China

**Keywords:** acute myeloid leukemia, allogeneic hematopoietic stem cell transplantation, immune reconstitution, osseous relapse, osteitis, SAPHO syndrome

## Abstract

**Background:**

New bone pain and destructive skeletal lesions after allogeneic hematopoietic stem cell transplantation (allo-HSCT) raise concern for malignant relapse or infection. Synovitis, acne, pustulosis, hyperostosis, and osteitis (SAPHO) syndrome is an uncommon inflammatory disorder that may produce overlapping imaging findings.

**Case presentation:**

A 19-year-old man with acute myeloid leukemia underwent haploidentical allo-HSCT in second complete remission. Thirteen months later, while measurable residual disease remained undetectable, he developed severe thoracolumbar pain and extensive inflammatory acneiform eruptions. Magnetic resonance imaging showed abnormal thoracic vertebral marrow and paravertebral signals, initially raising suspicion of osseous leukemic relapse. C-reactive protein and erythrocyte sedimentation rate were elevated, whereas bone marrow examination confirmed continued remission. Computed tomography and technetium-99 m methylene diphosphonate bone scintigraphy with single-photon emission computed tomography/computed tomography demonstrated lesions involving the sternum, bilateral clavicles, and T6–T9 costovertebral joints. Computed tomography-guided sternal biopsy showed collagenized fibrous tissue with myxoid change, focal chondroid differentiation, a small amount of newly formed bone, and mild inflammatory-cell infiltration, without morphologic evidence of leukemic involvement. The combination of severe acne and osteitis in a characteristic distribution, after clinical evaluation for malignancy and infection, supported a diagnosis of SAPHO syndrome. Serum interleukin-6 elevation and altered peripheral immune-cell subsets were considered exploratory single-time-point observations. Symptoms improved after methylprednisolone followed by celecoxib, methotrexate, and dermatologic treatment.

**Conclusion:**

SAPHO syndrome should be considered when post-transplant bone lesions coexist with compatible skin manifestations. Multimodal imaging and targeted biopsy helped avoid inappropriate treatment for presumed AML relapse. The observed immune abnormalities are hypothesis-generating and do not establish a causal relationship between allo-HSCT and SAPHO syndrome.

## Introduction

SAPHO syndrome is an uncommon inflammatory disorder characterized by variable combinations of osteitis, hyperostosis, synovitis, severe acne, and palmoplantar pustulosis (1, 2). The anterior chest wall is the most frequently affected site; the spine, sacroiliac joints, and long bones may also be involved (2). No single laboratory test is diagnostic. Diagnosis therefore rests on the clinical pattern, imaging findings, and reasonable exclusion of infection and malignancy, with biopsy often required for atypical or destructive lesions (1–3).

New focal bone pain after allo-HSCT presents a high-stakes differential diagnosis. Malignant relapse, bacterial or fungal osteomyelitis, treatment-related injury, graft-vs.-host disease (GVHD)-associated musculoskeletal manifestations, and noninfectious inflammatory disease may overlap clinically and radiologically. Magnetic resonance imaging (MRI) is sensitive to marrow abnormalities but is not disease-specific (4). In a patient transplanted for acute myeloid leukemia (AML), destructive vertebral lesions may therefore prompt treatment for relapse before their cause has been established.

Autoimmune and inflammatory disorders may arise during immune reconstitution after allo-HSCT (5–7), but a temporal association does not establish that transplantation caused a subsequent inflammatory disease. To assess the novelty of this presentation, PubMed/MEDLINE and Google Scholar were searched from inception through 30 June 2026 using combinations of “SAPHO” or “synovitis acne pustulosis hyperostosis osteitis” with “allogeneic hematopoietic stem cell transplantation,” “hematopoietic stem cell transplantation,” “bone marrow transplantation,” “acute myeloid leukemia,” “leukemia,” “malignancy,” and “cancer.” No previous report of SAPHO arising after allo-HSCT was identified. The closest oncologic precedent was the case reported by Kubo et al., in which SAPHO mimicked bone metastases during pembrolizumab treatment for non-small cell lung cancer (8). We report a case that presented 13 months after haploidentical allo-HSCT and initially mimicked osseous AML relapse. The case is reported in accordance with the CARE guideline (9).

## Case description

A 19-year-old man was diagnosed with AML in August 2024 (Figure 1). Bone marrow aspirate contained 79.5% myeloblasts. Molecular testing (CEBPA transcript NM_004364.4) identified c.934_936dupCAG (p.Gln312dup; VAF, 43.7%), an in-frame duplication in the bZIP region, and c.125dupC (p.Pro43fs; VAF, 40.6%), a frameshift variant outside the bZIP region. Variant phasing was not performed; therefore, whether the two CEBPA variants were in trans could not be determined. Additional variants involved STAT5B (VAF, 45.1%), TET2 (VAF, 49.0%), and WT1 (VAFs, 82.7% and 2.7%). He received idarubicin plus cytarabine induction and high-dose cytarabine-based consolidation. Hematologic relapse occurred in February 2025, with 8% marrow blasts and flow-cytometric measurable residual disease (MRD) of 3.24%. Salvage therapy with idarubicin, cytarabine, and venetoclax induced a second complete remission.

**Figure 1:**
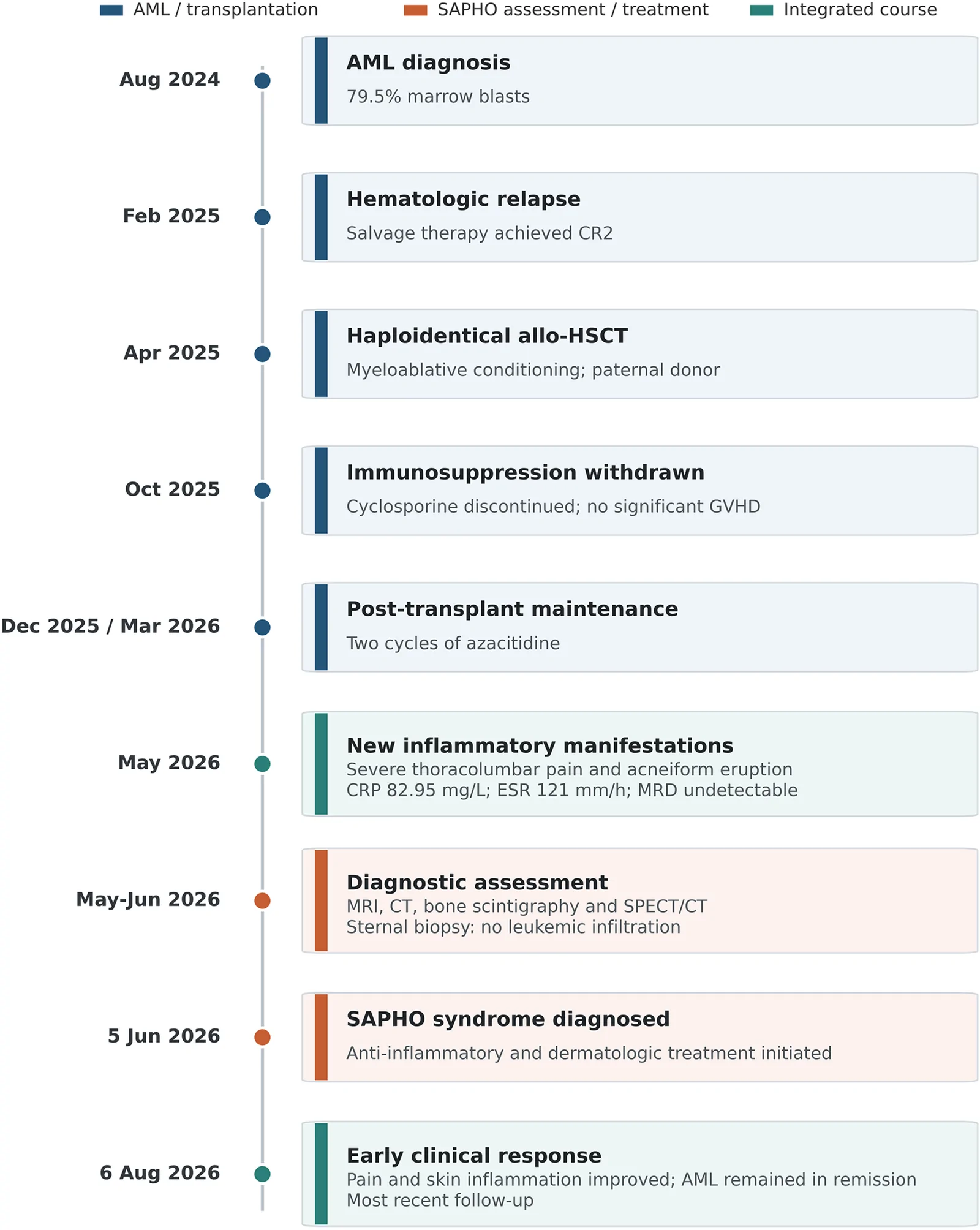
Clinical timeline from AML diagnosis through the most recent follow-up. AML, acute myeloid leukemia; allo-HSCT, allogeneic hematopoietic stem cell transplantation; CR2, second complete remission; CRP, C-reactive protein; ESR, erythrocyte sedimentation rate; MRD, measurable residual disease; SAPHO, synovitis, acne, pustulosis, hyperostosis, and osteitis; SPECT/CT, single-photon emission computed tomography/computed tomography.

In April 2025, he underwent myeloablative haploidentical allo-HSCT from his father (HLA 5/10 matched; donor blood group B positive and recipient AB positive). Conditioning comprised cytarabine, busulfan, cyclophosphamide, and semustine. GVHD prophylaxis comprised antithymocyte globulin, cyclosporine, mycophenolate mofetil, and short-course methotrexate. Neutrophil and platelet engraftment occurred on days +19 and +24, respectively. Cyclosporine was tapered and discontinued in October 2025. He developed no clinically significant acute or chronic GVHD. Two cycles of azacitidine maintenance were administered in December 2025 and March 2026. Serial marrow examinations showed continued morphologic remission with undetectable MRD. He had no history of acne, palmoplantar pustulosis, inflammatory arthritis, or chronic bone pain before AML.

There was no relevant family history of hematologic malignancy, inflammatory bone disease, severe acne, or psoriasis. He did not smoke or drink alcohol and worked in the catering industry. No clinically relevant psychosocial or mental-health concerns were reported.

In May 2026, 13 months after allo-HSCT, the patient developed progressively severe thoracolumbar pain that limited daily activities. At approximately the same time, inflammatory papules and pustules appeared extensively on the face (Figure 2). His white blood cell count was 9.34 × 109/L, neutrophil count 6.97 × 109/L, hemoglobin 124 g/L, and platelet count 210 × 109/L. C-reactive protein was 82.95 mg/L and erythrocyte sedimentation rate was 121 mm/h. Renal and hepatic biochemical tests were unremarkable. HLA-B27, rheumatoid factor, and anti-cyclic citrullinated peptide antibodies were negative; anti-PM-Scl was equivocal.

**Figure 2:**
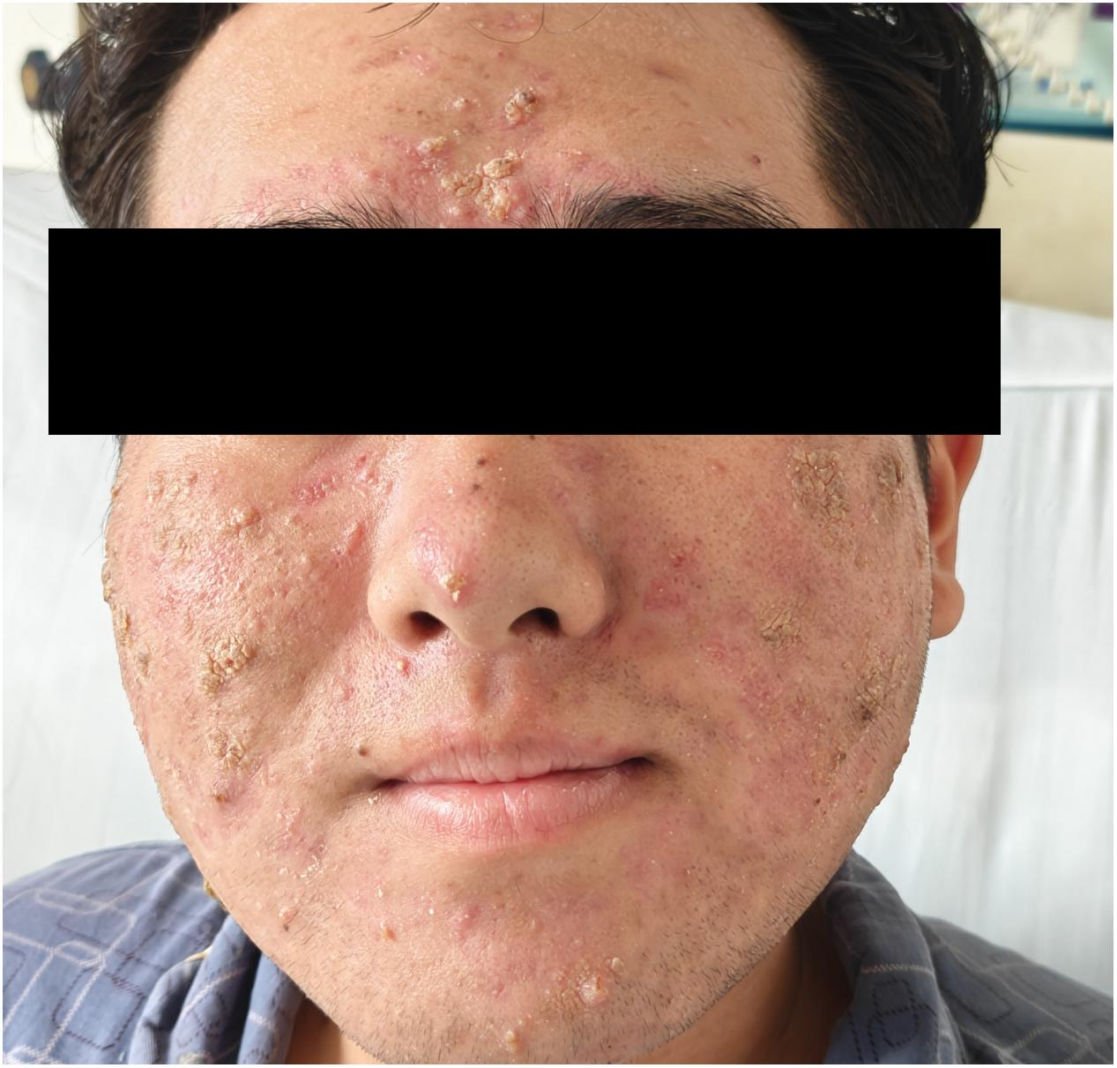
Cutaneous manifestations at presentation. Widespread inflammatory acneiform papules, pustules, crusted erosions, and diffuse erythema involving the forehead, cheeks, and nasal bridge before anti-inflammatory and dermatologic treatment.

Serum (1, 3)-β-D-glucan and galactomannan assays, plasma Epstein–Barr virus DNA, and T-SPOT.TB were negative. No bloodstream pathogen was identified. Culture of material from an acne pustule grew Serratia marcescens. Because no bacteremia or systemic infectious syndrome was present, this superficial culture was not considered sufficient evidence of S. marcescens osteomyelitis. The result was interpreted in conjunction with imaging, pathology, and the clinical course. The biopsy was performed primarily to evaluate suspected osseous AML relapse; because the patient had no fever, bacteremia, or systemic infectious syndrome at the time of sampling, allocation of fresh tissue for microbiological culture or molecular testing was not prospectively planned. This was an important diagnostic limitation in an allo-HSCT recipient, and an indolent bone infection could not be completely excluded.

Spinal MRI demonstrated abnormal marrow signals in the T6–T9 vertebral bodies with adjacent paravertebral soft-tissue signal abnormalities, raising concern for leukemic infiltration or infection (Figure 3A). Bone marrow aspirate showed continued morphologic remission and MRD remained undetectable; these findings reduced but did not independently exclude an isolated osseous relapse. Thoracic computed tomography (CT) showed destructive changes involving the thoracic vertebrae and abnormalities around both sternoclavicular joints (Figure 3B).

**Figure 3:**
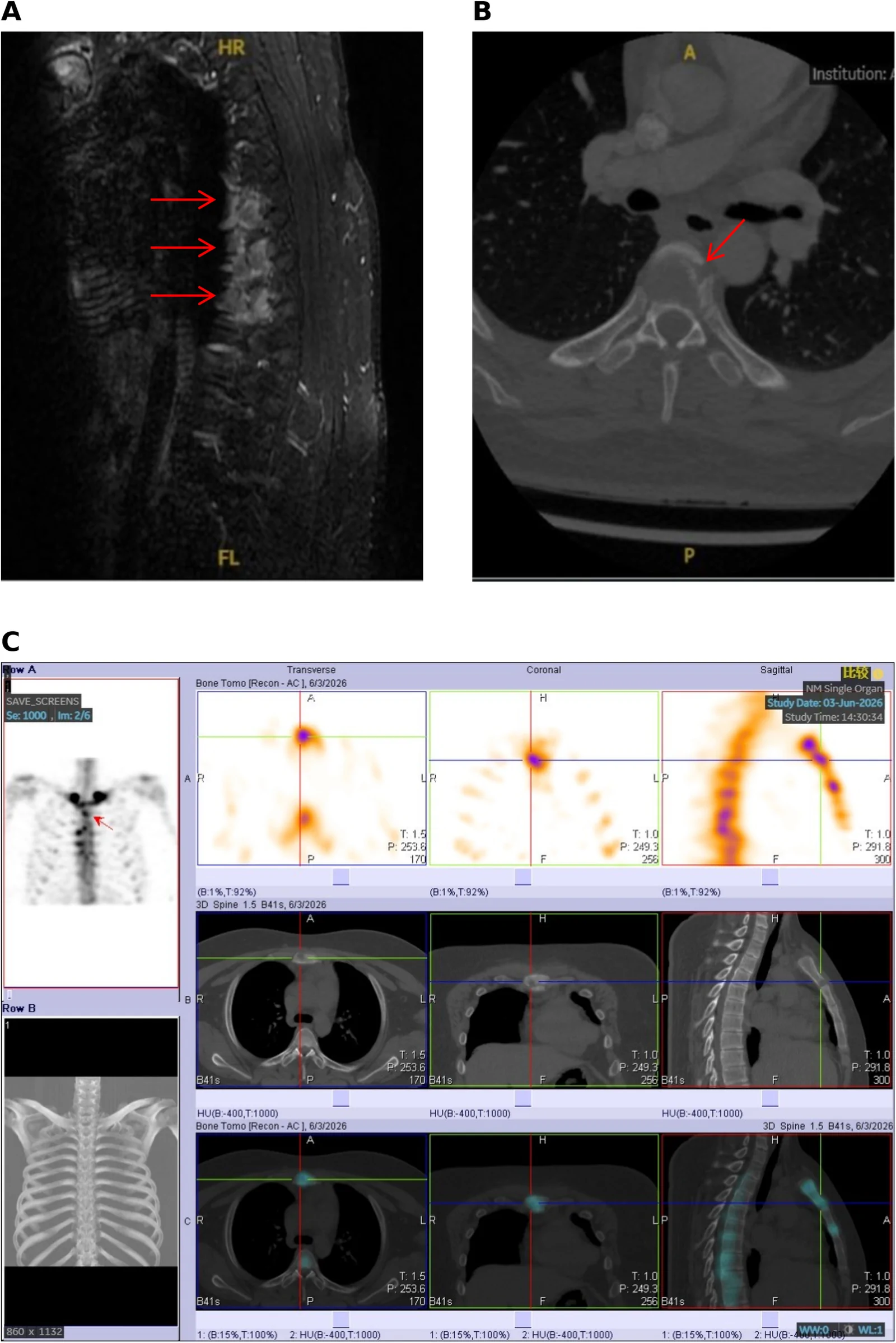
Multimodal imaging assessment of the skeletal lesions. **(A)** Sagittal spinal MRI showing abnormal marrow signal in the T6–T9 vertebral bodies with adjacent paravertebral soft-tissue signal abnormality (red arrows). **(B)** Axial thoracic CT showing an osteolytic vertebral lesion (red arrow). **(C)** Representative 99mTc-MDP bone scintigraphy and regional SPECT/CT display. The planar and SPECT images show prominent radiotracer uptake centered on the anterior chest wall; the corresponding low-dose CT and fused images anatomically localize the metabolically active lesion. The complete nuclear medicine examination additionally demonstrated increased uptake in the bilateral clavicles, sternum, and bilateral T6–T9 costovertebral joints, as described in the text.

Whole-body technetium-99 m methylene diphosphonate (99mTc-MDP) bone scintigraphy demonstrated abnormally increased radiotracer uptake in the bilateral clavicles, sternum, and approximately T6-T9 levels. Regional SPECT/CT further localized the increased uptake to the sternal ends of both clavicles, the sternum, and the bilateral T6-T9 costovertebral joints, with greater uptake on the right. Quantitative SPECT standardized uptake values and a prespecified semi-quantitative uptake score were not acquired; interpretation was therefore qualitative. Representative SPECT, low-dose CT, and fused SPECT/CT images demonstrated prominent uptake centered on the anterior chest wall (Figure 3C). The nuclear medicine interpretation favored inflammatory skeletal lesions and recommended follow-up imaging. In conjunction with the characteristic anterior chest-wall involvement and inflammatory acneiform eruptions, these findings raised clinical suspicion of SAPHO syndrome.

Given the patient's AML history and the destructive osseous changes, the sternal lesion was selected for CT-guided core biopsy. Histopathologic examination revealed proliferative fibrous tissue with collagenization and myxoid change, accompanied by focal chondroid differentiation and a small amount of newly formed bone (Figures 4A,B). Proliferation of thin-walled small vessels was observed, with focal mild infiltration by lymphocytes and eosinophils; a small focus of stratified squamous epithelium was also present (Figure 4C). No morphologic evidence of leukemic infiltration was identified in the submitted tissue. Because bone tissue was not submitted for microbiological culture or broad-range 16S rRNA sequencing, an indolent infectious process could not be completely excluded microbiologically.

**Figure 4:**
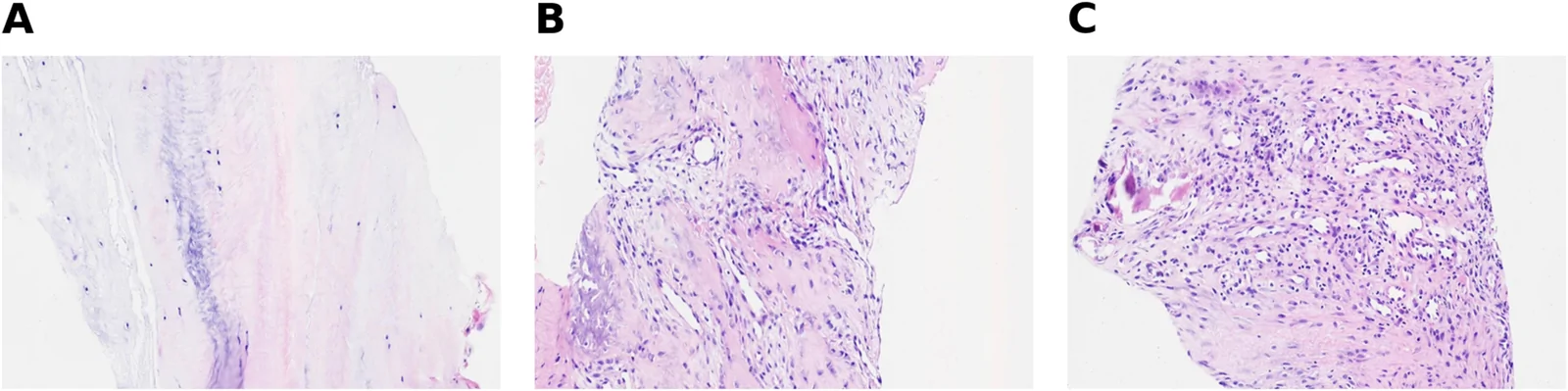
Histopathologic findings of the CT-guided sternal core biopsy. Hematoxylin and eosin staining, original magnification, ×20. **(A)** Focal chondroid differentiation within collagenized fibrous tissue with myxoid change. **(B)** A small amount of newly formed bone within the fibrous and myxoid stroma. **(C)** Proliferation of thin-walled small vessels with focal mild lymphocytic and eosinophilic infiltration. A small focus of stratified squamous epithelium was also present. No morphologic evidence of leukemic infiltration was identified.

The patient fulfilled commonly used diagnostic criteria for SAPHO syndrome through the association of severe acne with osteitis in a characteristic distribution, after reasonable clinical exclusion of malignancy and active infection (10, 11). Chronic nonbacterial osteomyelitis, infectious osteomyelitis, axial spondyloarthritis, chronic GVHD, and osseous AML relapse were considered. The patient's age and multifocal osteitis overlapped with chronic nonbacterial osteomyelitis; however, the combination of severe acne and characteristic anterior chest-wall involvement favored the SAPHO spectrum (1, 2). The absence of sacroiliitis and negative HLA-B27 testing did not support axial spondyloarthritis. The absence of prior GVHD and of characteristic cutaneous, oral, hepatic, or pulmonary GVHD manifestations made chronic GVHD less likely.

A multiplex serum cytokine panel obtained during active symptoms showed elevated IL-6 (18.99 pg/mL; laboratory reference interval, 0-5.4 pg/mL). The other 11 measured cytokines (IL-5, IFN-α, IL-2, IL-1β, IL-10, IFN-*γ*, IL-8, IL-17, IL-4, IL-12p, and TNF-α) were within their respective laboratory reference intervals (Supplementary Table S1). Flow cytometry showed a CD4 + effector T-cell proportion of 7.09% (reference interval, 0.44%–6.06%), regulatory T cells of 5.94%, CD8 + CD27- cells of 66.32%, CD8 + CD28- cells of 86.43%, and CD8 + central memory T cells of 2.10%. Myeloid dendritic-cell subsets were reported as MDC1 71.79% and MDC2 15.38%. Absolute counts were not available; all immune-cell results are therefore presented as proportions only.

These results were generated from a single sample without a pre-symptom specimen, post-treatment sample, or matched allo-HSCT control. The laboratory reference intervals were derived from non-transplant adults. The findings therefore describe immune status at presentation but cannot distinguish SAPHO-associated changes from the effects of conditioning, antithymocyte globulin, prior immunosuppression, infection exposure, or post-transplant immune reconstitution.

Intravenous methylprednisolone 40 mg/day was initiated on 5 June 2026 and produced rapid improvement in pain and inflammatory symptoms. Oral methylprednisolone 20 mg/day was started on 9 June 2026, reduced by 4 mg every 7 days (20 mg/day on 9-15 June, 16 mg/day on 16-22 June, and 12 mg/day on 23-29 June), then maintained at 8 mg/day from 30 June through 29 July and reduced to 4 mg/day from 30 July. The patient was still receiving 4 mg/day at the last follow-up. Celecoxib 200 mg/day and methotrexate 10 mg/week were prescribed for ongoing musculoskeletal disease. Minocycline 100 mg/day and topical metronidazole twice daily were used for the facial eruption. At the last evaluation on 6 August 2026, 62 days after initiation of SAPHO-directed therapy, the pain score had improved to 1 on a 0-10 numerical rating scale and CRP had decreased from 82.95 to 15.6 mg/L; ESR was not repeated. Facial skin inflammation was clinically documented as improved, although no standardized dermatologic score or follow-up photograph was available. No repeat MRI, CT, bone scintigraphy, or SPECT/CT was performed. Medication adherence was good, treatment was tolerated, and no adverse events were reported. AML remained in morphologic remission with undetectable MRD.

## Discussion

This case illustrates a diagnostically important mimic of post-transplant AML relapse. The diagnosis did not depend on MRI alone. Continued marrow remission, the combination of severe acne and anterior chest-wall osteitis, the distribution on bone scintigraphy and SPECT/CT, and the absence of malignant cells in a targeted bone biopsy collectively supported SAPHO syndrome. Tissue sampling was particularly important because isolated extramedullary relapse may occur despite a negative marrow examination.

Kubo et al. similarly described SAPHO mimicking malignant skeletal disease during pembrolizumab therapy for non-small cell lung cancer (8). Both cases illustrate how inflammatory osteitis can be mistaken for malignant bone involvement; however, the present case arose after allo-HSCT and mimicked osseous AML relapse, a post-transplant setting not identified in our literature search.

SAPHO lesions commonly involve the sternoclavicular region and sternum, and spinal disease may manifest as vertebral marrow edema, endplate abnormalities, paravertebral inflammation, osteitis, or hyperostosis (2, 3, 12). These findings can resemble infection, spondyloarthritis, or malignancy. The classic “bull's head” appearance on bone scintigraphy is suggestive when present but is neither invariably present nor sufficient to establish the diagnosis (12). Histopathology is also not pathognomonic; its appearance varies with disease stage and may range from acute inflammation to chronic fibro-osseous change (1, 2). In this patient, the biopsy was used primarily to assess leukemic involvement and characterize the lesion rather than as a stand-alone positive test for SAPHO syndrome.

The isolation of *S. marcescens* from an acne pustule requires cautious interpretation. Cutaneous lesions may be colonized or secondarily infected, whereas SAPHO-associated osteitis is generally considered noninfectious. Factors arguing against an invasive bone infection included the absence of bacteremia or systemic infectious manifestations, the characteristic clinicoradiologic distribution, and clinical improvement without prolonged pathogen-directed antimicrobial therapy. Nevertheless, because bone tissue was not cultured or analyzed by molecular microbiological methods, an indolent infection cannot be excluded with absolute certainty. This limitation is especially relevant in a recipient of allo-HSCT.

The temporal relationship between allo-HSCT and SAPHO syndrome is notable but does not prove that transplantation triggered the disease. Immune recovery after allo-HSCT is prolonged and nonuniform. Early expansion of mature donor T cells, delayed thymus-dependent production of naïve T cells, conditioning-related tissue injury, infections, and immunosuppressive therapy may all influence T-cell composition (6, 7, 13). CD28-negative and CD27-negative CD8 + populations may expand after repeated antigenic stimulation and during post-transplant immune remodeling; they should not automatically be interpreted as pathogenic senescent cells without functional assays (14). Likewise, IL-6 is a nonspecific inflammatory mediator that has been reported in SAPHO syndrome and other inflammatory states (15).

The immune findings in this case are therefore hypothesis-generating. They are compatible with post-transplant immune remodeling during an inflammatory episode, but they cannot demonstrate that IL-6, CD8 + differentiation, or dendritic-cell alterations caused SAPHO syndrome. Longitudinal sampling before and after clinical improvement, absolute cell counts, complete assay definitions, and comparison with allo-HSCT controls would be required to evaluate such a mechanism. A single patient's values cannot define a transplant-specific SAPHO immune signature.

Evidence guiding SAPHO treatment is based predominantly on observational studies. Nonsteroidal anti-inflammatory drugs are commonly used initially; glucocorticoids, conventional disease-modifying antirheumatic drugs, bisphosphonates, and targeted biologic agents have been used for refractory disease (2, 16). In a transplant recipient, management must also account for infection risk, marrow function, drug interactions, and relapse surveillance. The favorable early response in this patient supports the working diagnosis but does not establish comparative treatment efficacy.

This report has several strengths. It documents a clinically important mimic of post-transplant AML relapse; integrates cutaneous findings, marrow assessment, MRI, CT, bone scintigraphy/SPECT/CT, and targeted histopathology; and presents the differential diagnosis and uncertainty explicitly. Targeted biopsy helped prevent premature attribution of destructive skeletal lesions to leukemia and potentially inappropriate salvage therapy. The report also avoids inferring causality from the temporal association between allo-HSCT and SAPHO syndrome. Limitations include the single-patient design and short follow-up; the absence of serial immune measurements, absolute immune-cell counts, and an allo-HSCT comparator; use of non-transplant adult reference intervals; and lack of quantitative SPECT metrics or follow-up imaging. Bone tissue was not submitted for culture or molecular microbiological testing, so indolent infection cannot be completely excluded. The immune findings are therefore descriptive and hypothesis-generating, and the early clinical response cannot establish comparative treatment efficacy.

## Conclusion

SAPHO syndrome is a relevant differential diagnosis when inflammatory acne is accompanied by destructive or metabolically active bone lesions after allo-HSCT. In this patient, multimodal imaging identified a characteristic distribution and targeted biopsy found no leukemic infiltration, allowing anti-inflammatory treatment rather than salvage chemotherapy. The single-time-point cytokine and immune-cell abnormalities should be treated as exploratory observations. Additional cases with longitudinal and controlled immune profiling are required before a causal relationship between post-transplant immune reconstitution and SAPHO syndrome can be proposed.

## Patient perspective

The severe back pain and facial skin lesions substantially affected my daily activities and caused considerable anxiety. Because of my previous acute myeloid leukemia, I was particularly concerned that the bone abnormalities represented disease relapse. The diagnostic process, including multiple imaging examinations and the bone biopsy, was stressful, but I understood that these investigations were necessary to determine the cause of the lesions. I felt relieved when the biopsy showed no evidence of leukemic involvement and the diagnosis of SAPHO syndrome was made. My back pain and skin inflammation improved markedly after treatment, and I gradually resumed my usual daily activities. I hope that sharing my experience may help clinicians and other patients recognize that new bone lesions after transplantation do not always indicate leukemia relapse.

## Data Availability

The original contributions presented in the study are included in the article/Supplementary Material, further inquiries can be directed to the corresponding authors.

## References

[1] Nguyen MT Borchers A Selmi C Naguwa SM Cheema G Gershwin ME. The SAPHO syndrome. Semin Arthritis Rheum. (2012) 42:254–65. 10.1016/j.semarthrit.2012.05.006 23153960

[2] Liu S Tang M Cao Y Li C. Synovitis, acne, pustulosis, hyperostosis, and osteitis syndrome: review and update. Ther Adv Musculoskelet Dis. (2020) 12:1759720X–20912865. 10.1177/1759720X20912865 PMC7236399 32523634

[3] Przepiera-Będzak H Brzosko M. SAPHO Syndrome: pathogenesis, clinical presentation, imaging, comorbidities and treatment: a review. Postepy Dermatol Alergol. (2021) 38:937–42. 10.5114/ada.2020.97394 PMC8802951 35125997

[4] Steinbach LS. MRI In the detection of malignant infiltration of bone marrow—a commentary. AJR Am J Roentgenol. (2007) 188:1443–5. 10.2214/AJR.06.1531 17515361

[5] Holbro A Abinun M Daikeler T. Management of autoimmune diseases after haematopoietic stem cell transplantation. Br J Haematol. (2012) 157:281–90. 10.1111/j.1365-2141.2012.09070.x 22360687

[6] Ogonek J Kralj Juric M Ghimire S Varanasi PR Holler E Greinix H. Immune reconstitution after allogeneic hematopoietic stem cell transplantation. Front Immunol. (2016) 7:507. 10.3389/fimmu.2016.00507 27909435 PMC5112259

[7] Krenger W Blazar BR Holländer GA. Thymic T-cell development in allogeneic stem cell transplantation. Blood. (2011) 117:6768–76. 10.1182/blood-2011-02-334623 21427289 PMC3128475

[8] Kubo Y Ito K Fujiwara Y Yoshida T Kusumoto M. Case report: sAPHO syndrome mimicking bone metastases during treatment with pembrolizumab for non-small cell lung cancer. Front Med (Lausanne). (2021) 8:679111. 10.3389/fmed.2021.679111 34368186 PMC8339402

[9] Riley DS Barber MS Kienle GS Aronson JK Von Schoen-Angerer T Tugwell P. CARE Guidelines for case reports: explanation and elaboration document. J Clin Epidemiol. (2017) 89:218–35. 10.1016/j.jclinepi.2017.04.026 28529185

[10] Kahn MF Khan MA. The SAPHO syndrome. Baillieres Clin Rheumatol. (1994) 8:333–62. 10.1016/S0950-3579(94)80022-7 8076391

[11] Benhamou CL Chamot AM Kahn MF. Synovitis-acne-pustulosis hyperostosis-osteomyelitis syndrome (SAPHO): a new syndrome among the spondyloarthropathies? Clin Exp Rheumatol. (1988) 6:109–12. 2972430

[12] Depasquale R Kumar N Lalam RK Tins BJ Tyrrell PNM Singh J. SAPHO: what radiologists should know. Clin Radiol. (2012) 67:195–206. 10.1016/j.crad.2011.08.014 21939963

[13] Storek J Geddes M Khan F Huard B Helg C Chalandon Y. Reconstitution of the immune system after hematopoietic stem cell transplantation in humans. Semin Immunopathol. (2008) 30:425–37. 10.1007/s00281-008-0132-5 18949477

[14] Mou D Espinosa J Lo DJ Kirk AD. CD28 Negative T cells: is their loss our gain? Am J Transplant. (2014) 14:2460–6. 10.1111/ajt.12937 25323029 PMC4886707

[15] Przepiera-Będzak H Fischer K Brzosko M. Serum IL-6 and IL-23 levels and their correlation with angiogenic cytokines and disease activity in ankylosing spondylitis, psoriatic arthritis, and SAPHO syndrome. Mediators Inflamm. (2015) 2015:785705. 10.1155/2015/785705 PMC4539212 26339141

[16] Wang Y Gu M Zheng Z Jiang H Han L Huang H. Therapeutic approaches for SAPHO syndrome from the perspective of pathogenesis: a review of the literature. Front Immunol. (2025) 16:1560398. 10.3389/fimmu.2025.1560398 40303415 PMC12037609

