# Supplementary material for: Case Report: SAPHO syndrome mimicking osseous relapse after haploidentical allogeneic hematopoietic stem cell transplantation for acute myeloid leukemia
Source: Front Med (Lausanne). 2026 Sep 28;13:1936597. doi: 10.3389/fmed.2026.1936597 (PMC13654757; doi:10.3389/fmed.2026.1936597)
Supplement: Supplementary file 1 [file Table1.docx]

Supplementary Table S1. Complete serum cytokine panel obtained during active symptoms.

| Cytokine | Value (pg/mL) | Reference interval (pg/mL) | Interpretation |
| --- | --- | --- | --- |
| IL-5 | 2.29 | 0-5.2 | Within range |
| IFN-α | 1.02 | 0-8.5 | Within range |
| IL-2 | 1.24 | 0-7.5 | Within range |
| IL-6 | 18.99 | 0-5.4 | Elevated |
| IL-1β | 2.32 | 0-12.4 | Within range |
| IL-10 | 2.1 | 0-12.9 | Within range |
| IFN-γ | 9.4 | 0-23.1 | Within range |
| IL-8 | 2 | 0-20.6 | Within range |
| IL-17 | 0.92 | 0-21.4 | Within range |
| IL-4 | 0.87 | 0-8.56 | Within range |
| IL-12p | 0.96 | 0-3.4 | Within range |
| TNF-α | 2.22 | 0-16.5 | Within range |

IL, interleukin; IFN, interferon; TNF, tumor necrosis factor.
